# Site of Resistance or Apparatus of Acquiescence? Tactics at the Bakery

**DOI:** 10.1163/18763375-01002002

**Published:** 2018-08-02

**Authors:** José Ciro Martίnez

**Affiliations:** University of Cambridge

**Keywords:** Jordan, tactics, resistance, subsidies, food politics, neoliberalism, comparative politics

## Abstract

This article explores the importance and impact of a set of actions through which bakers manipulate laws and regulations that seek to organize and regulate how they do business. It builds on eighteen months of fieldwork conducted in Jordan, twelve of which were spent working in three different bakeries in the capital, Amman. Moving away from the idea that public policies are simply imposed, the article looks in detail at the social relations through which they are enacted. By honing in on the bakery, and examining arrangements between bakery owners, workers, consumers and ministerial employees, it illuminates modes of political agency that escape conventional binaries of domination/resistance, state/society and legality/illegality. I argue against seeing these practices as easily categorized forms of resistance or frivolous acts of corruption. Nor are they simply reinforcements of hegemonic control. Instead, ‘tactics’ at the bakery subvert the order of things to serve other ends. Foregrounding them in this analysis seeks not only to challenge views of power relations as strictly binary but to elucidate some of the ways in which citizens inhabit and engage with the neoliberal and authoritarian logics that pervade everyday life in Jordan.

At four-thirty in the morning, Hani ambles down the hill from his house in Jabal B, a poor neighborhood in East Amman.^1^ A five-minute walk takes him to his bakery. He turns on the lights, takes a quick glance at some paperwork, jump-starts the flour mixer and fires up the oven. By five, his workers have joined him. They drop their coats and belongings in the backroom and begin the day’s labor. One works the flour mixer, two prepare dough and three bake bread and ready it for sale. The same six men will work until nine at night, in two shifts interspersed by a two-hour lunch break. Hani spends most of the day at the cash register chain smoking. From his perch, he barks orders at the workers, greets customers amicably and sorts out the minutiae that make the business run: salt procurement, payment of electricity bills, taxation matters and the purchase of subsidized flour. It is the last of these tasks that consumes most of his time and causes the majority of his unease. The Ministry of Industry, Trade and Supply (MOITS) closely regulates the subsidized bread business. MOITS intervenes in all steps of the supply chain: it purchases wheat on international markets, oversees the production and delivery of discounted flour and regulates the price of subsidized bread sold to consumers. Bakeries both big and small such as Hani’s are given flour quotas which must, by law, be used exclusively for the production of standard *khubz ’arabl* (Arab—or pita—bread) that is sold at 0.16 JD (0.23 USD) per kilogram.^2^ Ministry employees set these quotas, which guarantee a 7% profit for bakers, through a complex and confidential formula based on a bakery’s potential output, the owner’s reputation and local demand. Once set, the quotas are very hard to change, especially when an increase is requested. MOITS employees, distraught by a thriving black market that drains government coffers, suspect bakery owners may re-sell their discounted flour or use it to make sweets, cakes and fancier varieties of bread, two common practices that are legally prohibited.

Three bakeries operate in Jabal B. One is smaller than Hani’s, with just two employees. Another closes at 3 in the afternoon, following the lunch rush. For local residents, many of whom work in the wealthier neighborhoods of West Amman, Hani’s outlet is the last place where they can buy bread for dinner. For others, it is the easiest place to obtain provisions for an early breakfast. The bakery’s proximity to the neighborhood mosque as well as its long opening hours contributes to its popularity. Hani sells only subsidized *khubz ’arabl.* He prefers not to prepare other, non-subsidized wheat-based products from which other bakeries derive most of their profits. His business practices target the local customer base: “Most families in this neighborhood are very poor. They may eat lamb or chicken once a week, they buy sweets in the *balad* (downtown) for holidays, but they eat bread with almost every meal,” he reasons. Since 2012, Jabal B has witnessed a sizeable influx of Syrian refugees, attracted by cheap rents and the neighborhood’s proximity to a major public transportation hub.^3^ Hani estimates that his customer base has grown by 15,000 people, yet his bakery has not been given a commensurate increase in its flour quota. This does not endanger his business—profits on subsidized bread sold from his quota remain steady at 7%—but it does threaten the routines, rhythms and subsistence of his customers; under his current flour quota subsidized bread provisions would be depleted by three or four in the afternoon, he estimates. Faced with this conundrum, Hani commits a crime. Once a week, he meets a ministerial employee and pays him a bribe that ensures supplementary deliveries of subsidized flour. Although arrangements such as these are frequently described as ‘corruption’ or a ‘failure of governance,’ they are better understood as ‘tactics’ that shape livelihoods in the city of Amman.

This article is based on a study of the Jordanian bread subsidy. It builds on eighteen months of fieldwork conducted in the country, twelve of which were spent working in three different bakeries in the capital, Amman.^4^ Here, I will explore the importance and impact of a set of actions through which bakers manipulate laws and regulations that seek to organize how they do business. A close study of the arrangements amongst bakery owners, workers, consumers and ministerial employees seeks to illuminate modes of political agency that escape conventional binaries of domination/resistance, state/society and legality/illegality that dominate the literature in comparative politics. I identify and analyze such arrangements by honing in on place. Specifically, this article examines the bakery as a salient sphere of everyday life, a crucial site in which state power, urban livelihoods and creative tactics intersect. Neither a hermetically sealed biophysical reality nor autonomous site of the subaltern, the bakery is a politically charged assemblage that is simultaneously conceived, lived and imagined.^5^ By conceptualizing place in this fashion, the article seeks to assess the constellation of situated everyday practices through which citizens navigate crosscutting fields of power. I argue against seeing these practices as easily categorized forms of resistance or frivolous acts of corruption or consumerism. Nor are they simply reinforcements of hegemonic control. Through the art of trickery, disruption and ingenuity, ‘tactics’ at the bakery subvert the order of things to serve other ends. Although the politics of these actions may be unclear, their tactical nature is both recognizable and effective. Foregrounding them in this analysis seeks not only to problematize prevalent dichotomies but to illuminate some of the ways in which citizens inhabit and engage with the neoliberal and authoritarian logics that pervade everyday life in Jordan.

## (Dis)Locating Grids of Rule

In the process of providing bread to its citizens, the Jordanian state must calculate and rationalize that which it distributes and those who receive it. To render this arena of intervention both technical and improvable, humans and things must be bounded, mapped, characterized and documented.^6^ Foucault terms the broader process in which such developments are enmeshed as the “governmentalization of the state”^7^—“the process by which the juridical and administrative apparatus of the polity come to incorporate the disparate arenas of rule concerned with the government of the population.”^8^ In Jordan as elsewhere, reference grids, censuses, and surveys seek to construct an abstract field of observation through which the governmental gaze can assess accurate information and travel without impediment.^9^ These “inscription devices”^10^ make information both knowable and quantifiable, they “render visible the space over which government is to be exercised.”^11^ By reducing the messiness of place and practice through standards and codifications, labels and texts, state actors can define problems, measure resources and specify sectors for intervention.^12^ yet the success of such efforts is far from certain. Forms of knowledge and technique are not the sole source of these failures; there are many reasons for such ruptures.^13^ Here I want to focus on the severe limits on governmental programs posed by their target—the dynamic forces of social life. Whether it be humans, things, environment or space, this “obdurate terrain” presents important constraints on that which governmental interventions seek to accomplish.^14^ Discrepancies between ambitions and outcomes traverse landscapes of rule; intended outcomes are never guaranteed.

Henri Lefebvre’s insights on the production of abstract space are productive for examining such discrepancies. His work considerably expanded the scope of Marxist sociology, and served to put the production of space at the forefront of considerations of capitalism and state power. Lefebvre posits that during the transition to capitalist modernity, the state apparatus produces a new form of socio-spatial organization, one characterized by its “ ‘abstract’ quality.” He terms this “abstract space,” a conceptualization that sees space as a solid, measurable substance, “an objectified, reified *thing*” (italics in original).^15^ Planners, engineers, developers and technocrats of all colors are crucial to abstract space’s circulation. It entails the classification and partitioning of political-economic life into clearly delineated jurisdictions that can be regulated and controlled. By codifying space into legible grids, characterized by fixity and homogeneity, abstract space attempts to inscribe ‘rational’ economic calculation in the spheres of production and exchange, as well as comprehensive control in the realm of statecraft.^16^ Such dynamics can be seen in the Jordanian government’s bread subsidy.

The Ministry of Industry, Trade and Supply (MOITS), which is charged with running the welfare program, seeks to organize, manage, standardize and control all aspects of the subsidized bread business. It fixes the country’s yearly wheat consumption, regulates the production of millers, supervises flour deliveries, sets profit margins for production and determines the nutritional needs of every single one of the country’s neighborhoods. It then uses this information to issue licenses to bakers, and establish their allocation of subsidized flour. Routine inspections of sanitary conditions, flour extraction rates and bread quality seek to ensure that regulations are observed and that standard *khubz ’arabl*is sold at the price of sixteen *qirsh* per kilogram. These efforts are more than public health or welfare monitoring measures. By constructing a governable grid out of complex irregularities, histories and practices, MOITS’S meticulous set of calculating practices, procedures and techniques seek to reinforce the state’s logic of socio-spatial control over humans and things.^17^ Lefe-bvre was hardly a proponent of this shift. He remained sharply critical of the technocratic and apolitical veneer through which abstract space was deployed and circulated, always emphasizing the inherently political nature of spatial organization. Crucially, Lefebvre’s analysis suggests that projects of state rationalization are not only fragmentary but also contested.^18^ Whereas the state apparatus attempts to remake space into ordered diagrams and governable grids, diverse social actors strive to create, preserve and expand places of social reproduction and grassroots control.^19^ The deployment of regulatory strategies does not necessarily imply their ultimate triumph. And it is the spontaneity of everyday life that remains a primary arena for prospective disruption, “the starting point for the realization of the possible.”^20^

## Practice and Politics in Everyday Life

Michel de Certeau’s work shares with Lefebvre an enduring interest in the everyday. While from a far different philosophical tradition, his examination of routine practices seeks to illustrate how users of space creatively manipulate mechanisms of discipline and homogenization. In *The Practice of Everyday Life,* de Certeau dissects the ways in which subjects of power play with the social order by using the very products and tools that order imposes on them, neither submitting to the demands of hegemonic forces nor confronting them head on. He conceptualizes the varied practices through which citizens and users reappropriate spaces organized and imposed by techniques of power as “tactics.”^21^ In so doing, he extends the domain of politics beyond formal institutions and collective mobilization to the diverse ways of operating within “the practice of everyday life.”^22^ Yet crucially, de Certeau’s story is not one of revolution or grand upheaval, but of stubbornness, obstinacy and creativity. Throughout his work, he stresses the innumerable constraints and the confined objectives of everyday practices. When “order is tricked by an art,” it is often done without any illusion that this order will change any time soon: “it is a maneuver ‘within an enemy’s field of vision’ and within enemy territory.’^23^ Bereft of the means to challenge a social order, tactics may deflect power or redirect its strictures, but always without leaving its field of operation.

Although a corpus of research on the politics of everyday life, largely inspired by James Scott,^24^ has drawn our attention to the various mechanisms through which subaltern and working classes engage with their socio-economic circumstances, these works far too frequently rely on dichotomous categories of domination and resistance. In the Middle East, everyday practices amongst popular classes have frequently been portrayed as defensive coping mechanisms or contentious acts of defiance that function as the building blocks of collective mobilization.^25^ The recent Arab uprisings do seem to indicate that these practices may have the potential to “become infrastructures of action— foundations upon which resistance in the form of collective action can be built,”^26^ but their importance is not exhausted by such possibilities. Time in the field allowed me to observe seemingly mundane actions that both express dissent but also employ calculated accommodation, that seek autonomy from oppressive governmental regulations while also demanding support from a beneficent state. They are neither revolutionary nor an indication of passivity. Of course, opposition and subversion are involved, but so are improvisation, negotiation with authorities and forms of “quiet encroachment”^27^ through which ordinary people attempt to improve their lives.^28^ By highlighting the routines, contestations and social relations in which these everyday practices are enmeshed, I seek to attend to the various ways in which citizens navigate the warrens of capitalist exchange and the grids of authoritarian rule, without succumbing to the “romance of resistance”^29^ or sanitizing the politics of sub-alterns.^30^ Melding Lefebvre’s meditations on abstract space with de Certeau’s notion of tactics, and drawing upon their shared concern with the everyday, I contend that, at the bakery, Jordanians encounter and confound the state apparatus’ regulatory techniques while establishing alternative modes of togetherness and consumption that make precarity livable.^31^

## Situating Tactics

Prior to the onset of structural adjustment measures in 1989, social welfare in Jordan was composed of an aggregate of public policies common to many “protective welfare states.”^32^ A curious fusion of Keynesian techniques employed for conservative ends, the system sought to acquiesce both labor and business while bolstering the Hashemite regime’s legitimacy. Generous public employment for East Bankers and universal consumer protections in the form of price supports and subsidies worked to shield families from labor market risk and economic downturns, all while lowering labor costs.^33^ These policies expanded substantially during the late 1970s. In addition to salary increases, government employees were given access to credit, health insurance and generous pensions. The population as a whole had access to basic commodities administered and regulated by the Ministry of Supply, formally established in 1974. Subsidized goods included: wheat, sugar, petroleum, powdered milk, tea, cigarettes and coffee. Over the next two decades, access to geopolitical and oil rents coupled with the Hashemite regime’s patrimonial tendencies led the state apparatus to become the main provider of crucial subsistence goods for many citizens. Although the country’s embrace of interventionist measures was not inspired by a coherent development model or Arab socialist ideology as in Egypt and Syria, welfare policies were slowly woven into a complex field of informal expectations amongst Jordanians who, criticisms notwithstanding, frequently refer to this period (1972-1988) as one characterized by job guarantees, access to affordable education and relative social equality.

The shift towards neoliberal economic policies engendered by the country’s debt crisis and the subsequent implementation of structural adjustment policies decreased welfare expenditures. Beginning in 1989, hiring in the public sector declined, except for the armed forces, and subsidies on most goods were eliminated. Price increases levied on fuel, residential water and various foodstuffs were enacted just as civil service pay was frozen,^34^ making it increasingly difficult for government employees to deal with inflation, which was rising by 30 to 50 percent at the time.^35^ Spending reductions in other social programs resulted in reduced access to education, health care, and affordable housing. These changes did not occur without social unrest or vociferous public protests. Increases in fuel prices in 1989 sparked immediate riots in the southern city of Ma’an, which quickly spread throughout the country. The removal of bread subsidies in 1996 ignited unrest in Karak, a town once considered a bastion of monarchical support.^36^ Those who fought to defend the provisionist status quo ante in the face of austerity measures imposed from above were able to extract occasional concessions^37^ Protestors and opposition parties set their critiques within the confines of established values and ideologies of the ruling order, which made them very hard to disavow^38^ These forms of “rightful resistance”^39^ won key victories, including increased participation in ostensibly democratic forums, discounted heating oil and the preservation of wheat and barley subsidies.

Despite these sporadic victories, the last two decades have negatively altered the living standards of Jordan’s poor and middle classes. Far from shielding them from the worst effects of austerity by way of private sector jobs, new business opportunities and access to financial tools, neoliberalization has worsened their plight.^40^ In the midst of booming service, construction and military sectors, inequality and poverty levels have worsened. Although official wages might be growing, employment is uncertain, and while taxes are comparatively modest, they remain regressive. The privatization of public companies, de-regulation of markets and implementation of free trade agreements have undoubtedly created new opportunities, but these have mainly benefitted a coterie of elites with close ties to the Palace^41^ Within this context of increasing inequality, social policy has seen a shift towards targeted anti-poverty schemes and cash transfers championed by international financial institutions. Initiated during the early 1990s, these programs have expanded under King Abdullah 11. Inadequately funded and administratively disjointed, observers have criticized these programs for their inability to reach all those in need, or to provide anything beyond basic assistance to the approximately 20 percent of the poor they successfully reach^42^ The re-structuring of the country’s welfare system paid little heed to the exigencies of its labor market, which is highly dependent on a service sector in which employment is seasonal, intermittent, and, quite often, informal.^43^ As a result, poor, working and middle-class sectors of society not connected to the military have been left to cobble together social welfare from NGOs, charities and kin mutual aid associations.

One key consequence of this reconfiguration is the increased vulnerability of poor and working classes to inflation and increases in the prices of subsistence goods. As Asef Bayat argues, this has caused a shift in popular needs and demands, not just in Jordan but also throughout the Middle East.^44^ Past struggles over wages and working conditions, the typical demands of trade unionism, have lost ground in favor of broader concerns related to issues of urban collective consumption—housing, electricity, water and food. Subsidized bread lies at the intersection of these developments. It is one of the few Jordanian welfare programs that have survived the erosion of social provisions that characterized the pre-austerity social contract and continues to represent a crucial hedge against inflation and volatile food markets for residents of the country. Yet the foodstuff’s importance transcends its financial value or nutritional properties; it is a cornerstone of urban and rural diets, a subsistence good through which feelings of groupness and community are (re) produced^45^Similar to corn in Mexico or the baguette in France, *khubz ’arabl* serves “as a centerpiece of daily ritual and social interaction,^46^ one that lies “at the core of both the material and symbolic organization of everyday existence.”^47^

Broadly popular and consistently defended, the bread subsidy also under-girds one of the most consequential and recurring encounters of ordinary citizens with the state apparatus—which seeks to meticulously control the production, distribution and consumption of *khubz ’arabl.* Yet this very monopoly periodically engenders inequitable outcomes, including neighborhoods where demand outstrips supply or vice versa^48^ In response, bakers and ordinary citizens employ various tactics so as to guarantee provision and ensure subsistence. Notwithstanding laws, regulations, sporadic edicts and routine inspections through which the bread subsidy is enacted, state strategies of regulation continuously produce their “own defiant outside.”^49^

## Jabal B: Bribing for Subsistence

Apart from Fridays, when the bakery opens after the weekly prayer, the routine at Hani’s locale is unwavering. Lunch plans vary, friends or spouses occasionally come by for brief conversation, fortnightly government inspections trigger a brief stoppage but during work hours, habits rarely fluctuate. Except for Saturdays between four and five in the afternoon. At this time, an acquaintance that I initially did not recognize arrives without fail. During my first weeks working at Hani’s, his weekly stopover caused an unease I could not grasp. Around thirty minutes before his expected arrival, Hani would take the old cigarette carton that functioned as the cash register into the backroom. He would emerge edgy and apprehensive, pace around nervously until the visitor appeared. The visitor skillfully managed the entire episode. He came in quietly, nodded at Hani, spent a few minutes whispering with him out of earshot in the backroom before walking briskly out of sight. In less than five minutes he was gone, folded bills tucked eagerly into his pocket.

Fahed was one of three MOITS employees charged with overseeing bakeries in East Amman. Following his fifth Saturday visit, I realized who he was, his casual *dishdasha* (kaftan) a stark contrast to his workweek attire of slacks and a buttoned down shirt. I had seen him during the routine inspections MOITS staff undertook at the bakery twice a month. After two months at Hani’s, I decided to ask the workers about the purpose of his visit. *“Fasdd* (corruption),” one worker told me, “ma*fesh ghayru”* (there is no alternative).” I asked them to explain but they demurred, probably uneager to share their employer’s business with an outsider whose presence they found puzzling. A month later, the routine still unchanged, I asked Hani about Fahed’s visits. *“Al-daf’a qabel al-raf’a* (pay to play),”^50^ he stated jokingly, brushing my inquiry off. After work ended that evening, Hani asked me to stay and offered to discuss my query.

“As you can tell, we barely have enough bread for all our customers,” Hani relayed after a few pleasantries, “and this has only gotten worse over the past three years.” I nodded in agreement, as he went on to detail the various occasions on which he had requested an increase in his flour quota from MOITS to no avail. “Every time they say no. We either do not have the baking capacity, or enough customers, or some other excuse,” he stressed. Hani then detailed how, for many of the neighborhood’s residents, survival or modest levels of subsistence would not be possible without the subsidized foodstuff they are able to purchase at his bakery, a reality that drove him to take the steps I had witnessed. He then expounded on the bribe itself, describing Fahed as an “old friend with a pure heart *(qalbu abyad)”* who understood “the situation,” as he lived in a nearby neighborhood. Irritated by the Ministry’s intransigence, which he traced to the neighborhood’s majority Palestinian population, support for Islamists and acute poverty, Hani had asked Fahed to arrange for supplementary deliveries of subsidized flour. He purchases at black market rates that leave him minimal profits but ensure he has enough to stay open thirteen hours a day.^51^

Hani professes to run his business in accordance with Islamic prescriptions, practicing commerce through a higher order of ethics characterized by modesty, thriftiness and abstention from superfluous profits. These traits were exemplified in his decision to pay higher wages to his workers and sell only subsidized bread, made strictly from discounted flour. During lengthy workdays, he would often cite a *hadith* (prophetic saying): “He is not a believer whose stomach is filled while his neighbor goes hungry.” When describing his business, he portrayed the bakery not simply as a workplace or profit source but as a sphere for moral yet effective economic purpose, a principled alternative to what he termed a nationally compromised state of affairs. Many local residents similarly described the bakery as a place somehow removed from the vicissitudes of the free market. “I feel safe at Hani’s,” one regular customer who I often conversed with explained, “The prices do not change. It is the one time in the day when I purchase something without pondering my budget or the difficulties of making ends meet.” His cousin Rashid, an employee at a nearby sandwich shop, similarly affirmed his attachments to place: “Hani makes money,” he reasoned, “but his bakery is not a regular business, just concerned with profit. We all feel like it is fixed part of our *hayy* (neighborhood). The owner cares for the customers, who rely on his bread to live.”

Of course, Jabal B’s residents are aware of the broader socio-spatial strategies that marginalize them; they are under no illusions about how or for whom the city works. Few of those I met had very radical positions, although many used religious ethics and modes of expression as a way to criticize an ostensibly rigged economic and political system seen to be led by corrupt elites.^52^ Their feelings of estrangement emerge from a sense that those in power work with a different set of values than their own, dedicated to profit-seeking capitalist operations rather than the public needs of the city’s inhabitants. Yet when faced with the slow erosion of their livelihoods, they invoke a language of connection, sentimentality and mutual dependence, attached to a densely affective place. They describe the bakery, much like Hani, as a site of pragmatic materiality, a node of nurturance that defies logics of “capital surplus absorption”^53^ that increasingly prevail in Amman. While many residents knew of or could surmise the extent of black-market operations that made Hani’s bread-baking possible, his noble aims coupled with widespread suspicion of the government not only precluded disapproval but garnered praise amongst his customers.^54^ A state of generalized corruption appeared to justify lesser illegalities. “We appreciate this place,” relayed one technician from a nearby garage who always purchased provisions for lunch, “We are thankful the owner does what he must to make our subsistence possible.” Hani’s success is judged neither by profit margins nor victories in a normative political sphere but rather by his deft management of what remains an extra-legal arrangement that, despite its criminalization, is not necessarily illegitimate.^55^

This baker’s business practices, especially his extensive opening hours and disinterest in engaging in other retail trades (sweets, expensive varieties of bread), are widely commended by local residents. Many described him as an exemplary business owner and generous man *(rajul karlm).* Yet to ensure his bakery’s success, Hani must cultivate social relations not only with Jabal B’s residents but also with ministry officials, negotiating fields of power as well as precise combinations of flour, water and salt. To do so, Hani asserts an abstract right to subsistence while paying a bribe to ensure its fulfillment. He adheres to governmental regulations but with a crucial transgression. His bakery exemplifies modes of economic action that evade the strict logics of the market while promoting an “ethics of care” for the community^56^ His everyday practices signal alternative modes of exchange that must navigate governmental regulations in order to manipulate them, all while responding to moral concerns and physical needs. Of course, his self-recognized ability to circumvent regulations does not mean he has overcome the system or secured his long-terms interests in perpetuity. He remains open to harassment, enforced closure or onerous fines. What Hani’s business practices do illustrate is how the MOITS’S attempts to impose abstract spatial grids inevitably encounter an urban landscape entangled in social relations, urban livelihoods and moral economies. Hani’s purposeful navigation of government regulations, here seen in the unofficial purchase of subsidized flour from a ministry employee, and the world of relations that give meaning to these dynamics, constitute the terms by which Hani disrupts the state’s regulatory techniques without challenging its order outright. This is the limber terrain on which government interventions and welfare policies are worked out. While state strategies may work through fictions of fixity, they always struggle with the ambiguities of tactical micro-practices.

## Jabal C: Sweetening Lives

**Figure 1:**
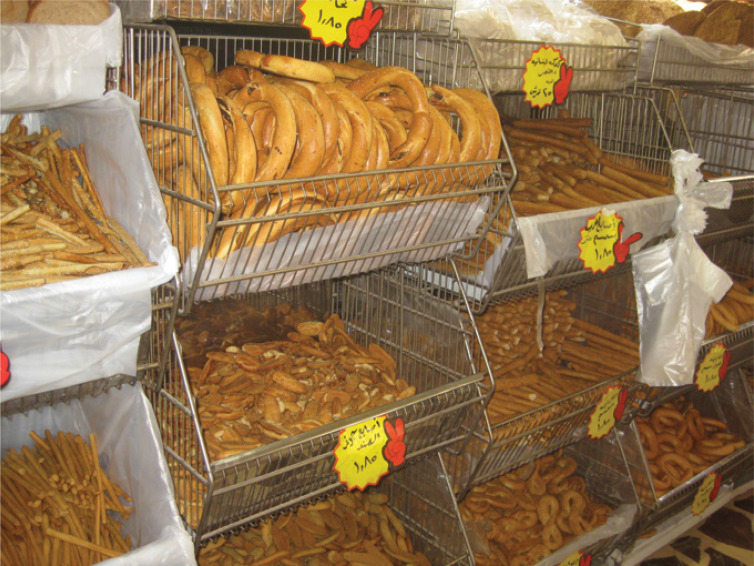
Varieties of ka'k

Jabal C is a lower-middle class neighborhood near Amman’s historic city center. It covers an area of 4 square kilometers and houses approximately 55,000 residents. The quarter began as an extension of a nearby Palestinian refugee camp in the early 1950s, but has expanded steadily. It is now a bustling commercial district. Local inhabitants work predominantly in the service sector. Small shopping malls, independent clothing shops, medical clinics, restaurants and other food purveyors are scattered throughout the neighborhood. While some families in the community are poor, most are lower or middle-class. Despite the disposable income and savings many possess, economic wellbeing is far from secure. Rising food and real estate prices are a source of constant worry and anxious conversation. I spent much of my time in Jabal C at al-Rif, one of six bakeries in this sub-district of Amman. In addition to standard *khubz ’arabl,* al-Rif sold non-subsidized *khubz ash-shrdk*^57^ a crucial ingredient the Jordanian national feast dish *mansaf*, ^58^ and an array of sweet biscuits (ka’k) that are usually dipped in tea (see figure 1). The latter, which come in various shapes and flavours, sold very poorly, a constant source of frustration for the bakery’s owner. Confident of the quality of his ka’k, he blamed dubious machinations at Samir’s, one of three nearby bakeries, for his bad luck.

**Figure 2:**
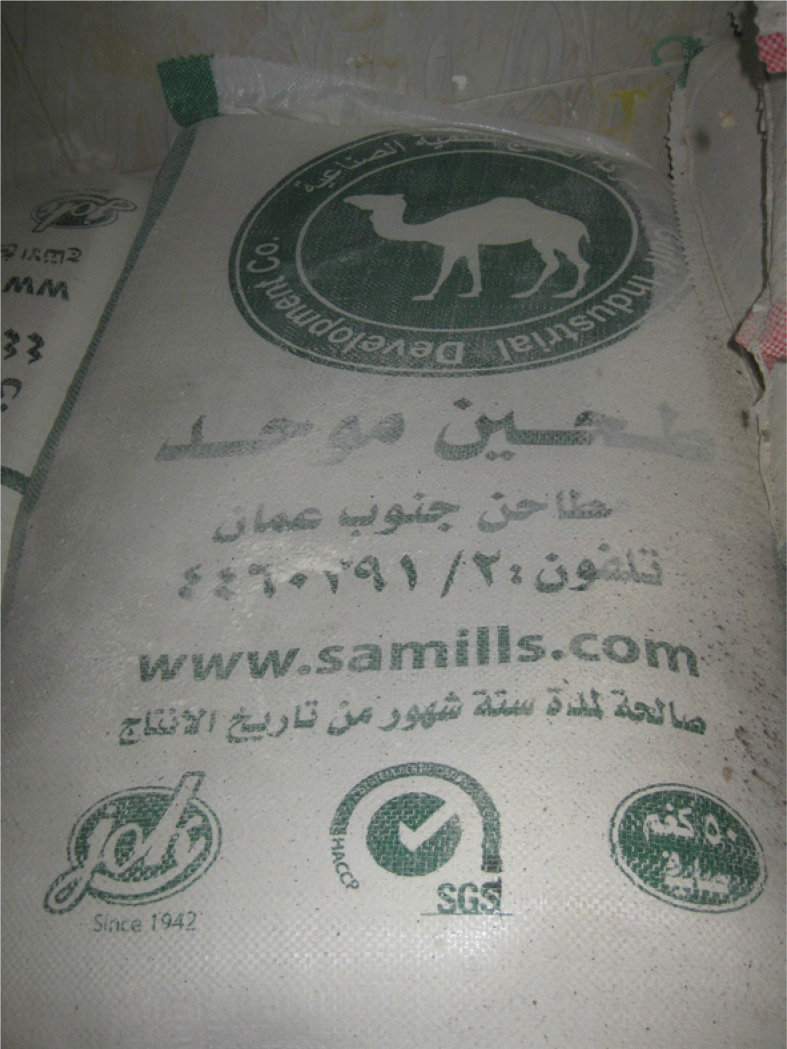
Subsidized Flour (Muwahhad)

**FIGURE 3:**
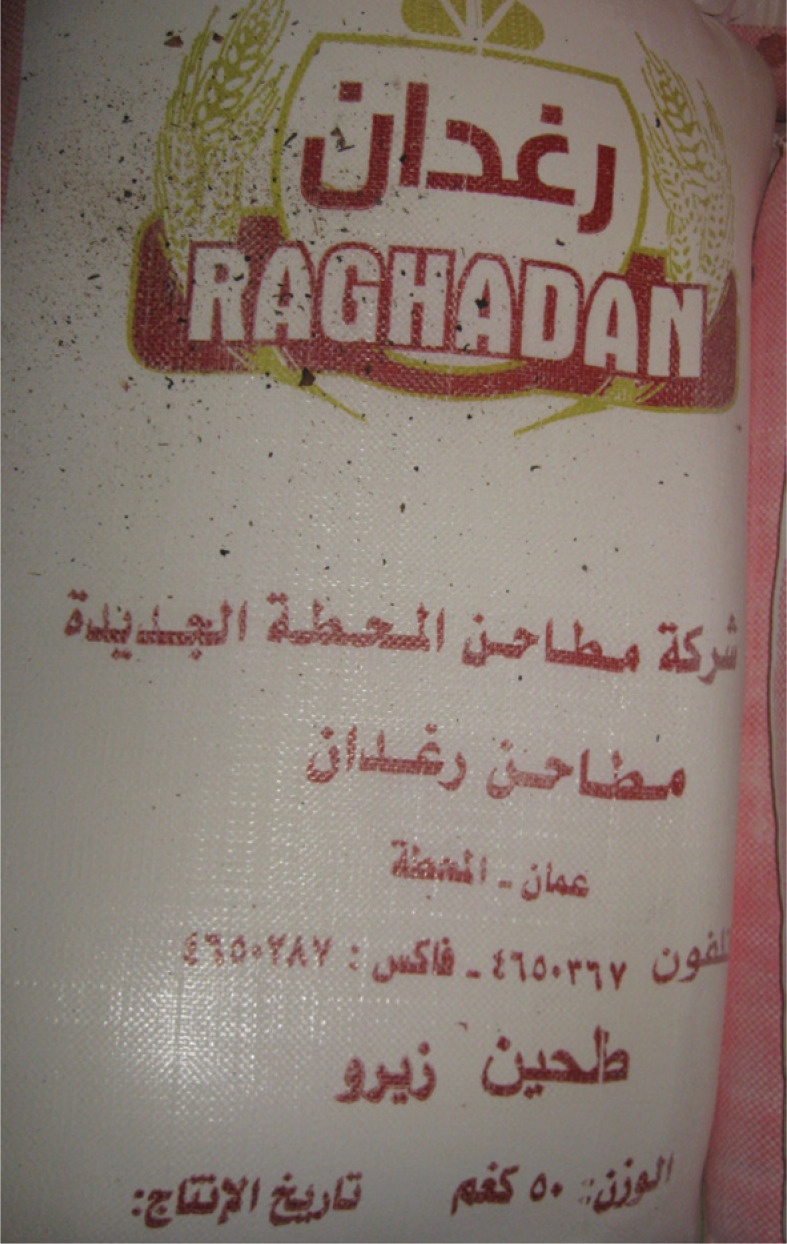
Non-subsidized flour (Zero)

After leaving work at al-Rif, I would often wander around the neighbourhood, shop for household supplies or join friends for coffee. One day, my curiosity piqued, I went to examine Samir’s baked goods. The reasons for his success selling biscuits quickly became apparent. Samir’s *ka’k* cost, on average, around half the price of its equivalents at other bakeries. My sponsor at al-Rif, wholly unsurprised by my finding, explained how this drastic markdown was achieved. He surmised that Samir made private use of a public resource by concealing its subsidized origins. He did so by shrewdly mixing subsidized flour *(al-Muwahhad),* with the non-subsidized variety (zero) typically used for biscuits (see figures 2 and 3).^59^ As the former cost one-seventh the price, Samir could sell *ka’k* at a dramatically lower price than his competitors while retaining a considerable profit margin^60^ Al-Rif’s owner accused Samir of “ihtiyal (fraud),” cheating both the government and his customers, who either could not tell the difference, or did not care given the price. His protestations to ministerial officials were frequent and heated. Partly due to such complaints, I was told that Samir’s bakery had been shut down on various occasions. Yet it always re-opened the next day, a product of his connections at the Ministry, al-Rif’s owner inferred. The story was more complicated.

I eventually met Samir through a common acquaintance at the Bakery Owner’s Association (BOA).^61^ He proved a stark contrast to the scheming caricature described at al-Rif. Exuberant and cheerful, his energy is infectious, a smile never too far from his face. His public self-presentation was that of a devout, God-fearing businessman, traits reflected in his place of business. His bakery was filled with Quranic suras, a sign behind his cash register read: *“Ahemiyyat al-khubzydti min al-Isldm* (The importance of bread comes from Islam).” The place was constantly bustling. Filled with customers throughout the day, he supplements retail transactions with a thriving wholesale trade with nearby hotels and restaurants. When explaining his success, Samir emphasized the social networks he had accumulated. He forges links with other merchants in activities such as raising money for local charities. On three separate evenings during Ramadan, I witnessed Samir and his business associates organize *Mawaid al-rahman* (tables of the Merciful), public banquets during which the poor are given food to break their fast, outside of his bakery. In addition to accumulating valuable symbolic capital, the social dimension of these acts helps develop ties between benefactors and recipients, which Samir would draw upon in key moments. Customers who I asked spoke of him highly, as did neighbouring business owners, who appreciate the footfall his bakery generates. When I eventually asked him about his *ka’k* prices, he was surprisingly forthcoming. Before 2008, flour was universally subsidized, he recounted accurately, the new legislation caused a huge price rise in all wheat-based goods except *khubz ’arabl*.^62^ While Samir saw no reason why croissants or fancy baguettes should receive government support, *ka’k* was different. “Cheap *ka’k* to dip with tea is hardly a luxury,” he stressed, “Enjoying these little pleasures offers a moment of respite during the long workday, or a small indulgence for poor families who could afford little else.” And crucially, his customers agreed.

During my time living in Amman, Samir’s bakery was closed on two occasions. Following complaints from anonymous members of the public, MOITS officials would arrive and inspect the extraction rate of various flour sacks and the quality of his baked goods. When inconsistencies were suspected, a MOITS official would meticulously catalogue their findings and take back samples to the head office for further inspection. After a weekly meeting at the Ministry in which violations were discussed, a committee would decree a temporary suspension in subsidized flour deliveries and issue a modest fine^63^ Samir’s response to this government intervention is tactical. He shuts his shop the same afternoon the decision is issued and posts a placard outside his business blaming the Ministry for the bakery’s closure. That evening, he mobilizes his networks for a protest. The following day, a crowd gathers on the street and sidewalks outside the bakery to the tune of various slogans revolving around bread. Hawkers, restaurants employees and off-duty construction workers in the vicinity were the main participants in the two protests I witnessed. Police had been notified of the gathering ahead of time and casually stood amongst the protestors. I watched uncomfortably as Samir approached them. He listed his complaints and explained the reasons for the protest. His remarks emphasized bread’s importance to the community, how the penalties endangered local residents’ access to a key subsistence good: “Our survival is at stake *(haydtuna ’ala-l-mlhak)”* and “our existence is in danger *(istimrdriyatuna fi khatar)“* were phrases he used repeatedly. Samir concluded by animating the crowd, which was now energetically waving loafs of bread.

To my surprise, the police were apathetic; two officers were holding back a smile. Notwithstanding the public spectacle, interactions with protestors were affable, even affectionate. The conversation that followed Samir’s remarks showed that their concern was not with the substance of his demands but with the logistics of the protest. Police officers explained that their job was to avoid traffic build-ups and violence amongst protestors. Much like the hawker protests Anjaria documents in Mumbai, remonstrations outside Samir’s bakery were “no dramatic challenge to authority but a cordial encounter.”^64^ This was neither a spontaneous expression of communal anger nor a typical opposition-led political rally, but a tactical medium used to communicate local grievances. Samir did not seek to contest or de-legitimize the Hashemite regime, which he openly supports and praises, but to mobilize the state apparatus to serve particular ends^65^

Once the press arrived, the gathering quickly dissipated. Demonstrators slowly dispersed and Samir huddled for a round of press interviews, some of which appeared in the nightly news or the next day’s newspaper. As Samir recounted, by seven or eight that evening he would receive a phone call from the liable Ministry official, who would duly inform him that the penalties imposed had been rescinded. “For the Ministry, it is not worth the bother,” he told me with a smile, “their job is to stay out of the news, they must have received twenty angry phone calls from powerful people yesterday.” Knowing this, Samir choreographed a protest to try and produce a desired outcome: he would notify the police, tipoff the press, who would make an appearance and transmit to the broader public the complaints aired in Jabal C. His success in having the fines annulled was not worked out in a juridical realm of rights or laws but in a domain grounded in everyday practices and relations.^66^ It is here, in this interstice, that the legitimacy of counterfeit *ka’k* is determined. Even though Samir’s *ka’k* remains illegal, his tactical response to MOITS penalties produces extralegal recognitions of his baking practices. His success illustrates how navigating government regulations involves not only balancing extraction rates and subsidized flour quotas but proficiency in “negotiating dense networks of mediation” that include Ministry officials, elected politicians and reliable customers^67^ The morning after the protest, Samir’s bakery was open and humming as usual. Governmental grids are always subject to tactical manipulations, forms of agency and inventiveness that make precarity livable.

I was struck by this event because of what it reveals about the possible kinds of engagements citizens have with state institutions. Through his astute and inventive use of crowd action, Samir marshals communal sentiments to convey local grievances and potential unrest. Yet his manoeuvre, as well as the protest outside his bakery, hardly qualifies as a straightforward moment of resistance. Far from contesting the legitimacy of the monarchy or the sovereignty of state institutions, his actions recognize the latter’s authority, their ability to adjudicate subsidies and impose penalties. Samir strives instead to manage the state’s performative interventions. His primary concern is to shape the relationship between the strictures of the law and its application at the bakery. His success, and the “generalized informal functioning” of government programs it indicates does not mark a complete absence of rules or laws, nor that his practices are merely a matter of regulatory expertise and personal connection^68^ On the contrary, Samir’s carefully choreographed social action is shaped by tacit codes and norms that he must carefully traverse. By making his claims on both practical and ethical grounds, Samir calls to account a state apparatus whose legitimacy partly rests on its commitment to “the people.”^69^ While the criminalization of Samir’s business practices may seem to close off certain possibilities, his skilled use of local networks make discounted biscuit sales possible. By tactically manipulating a realm of ordinary activity, and coding his protest with political meanings, he defies governmental regulations in a fashion that is awkward for the Hashemite regime to suppress. In this regard, Jordan’s bakers do not navigate a political sphere of rights-bearing citizens or the rational machinery of an anonymous bureaucracy. They operate within a supple terrain of illegalities where success means neither revolution nor social upheaval but, as Samir put it, the ability to “sweeten [people’s] lives.”

Widely denounced by government officials as “fraud” that attenuates against the whole subsidy system, Hani’s Saturday routine and Samir’s use of crowd action are much more than a perversion of how the subsidized bread welfare program ought to work. While the payment of bribes and the selective enforcement of regulations point to the presence of a predatory state, relationships and practices that emerge from such encounters also serve to open up possibilities. ‘Corruption’ here serves not only to alienate or disenfranchise citizens. Informal arrangements and personal relationships between low-level ministry employees and bakers enable substantive rights to bread to be negotiated and maintained, outside the proceduralist realm of the law.^70^ In the instances discussed herein, their interactions are not simply combative but collaborative;^71^ they involve moments of mediation where governmental grids and regulations are tactically manipulated. Such interactions make clear that policeman, ministerial employees, bakers, and ordinary citizens are not simply the autonomous agents of the state, business and society, but are likewise entangled in ongoing relations of kinship, friendship and community that engulf the governmental grids that seek to carve them up. Reducing such exchanges to a normative breakdown of governance or a corruption of public programs overlooks the compromises, mediations and tensions that characterize the citizenry’s everyday encounters with the state—a contingent formation whose apparent unity always condenses contradictions.^72^

## Conclusion: Agency in a Time of Constraints

Amman’s bakers epitomize many paradoxes: cherished providers, despised competitors, legitimized lawbreakers and precarious persistence. Their practices defy easy characterization and their relationship with state institutions is complex, characterized by predatory extortion, occasional collaboration, subtle negotiation and uneasy compromise. Or consider the bakery itself: while practices of planning see the bakery through the prism of abstract space: diagrams and statistics, all with objective properties, informal arrangements between low-level ministry officials and bakers reflect relational histories and subtle negotiations that occur in a shared social world.^73^ Despite clear differences in their formal relationships to structures of power and the hierarchies of class, gender and age that inflect their modes of interaction, certain forms of mediation and compromise are still possible. Systems of affinity are multiple and dense, few are unencumbered. This does not mean we should idealize the bakery as a subaltern venue for service provision or as an informal avenue of participation. Nor do I wish to posit practices at the bakery as coherent alternatives to the dominant order that can stand in somehow for radical ideological action.^74^ Indeed, I wish to warn against romanticizing the precarious condition Samir and Hani’s bakeries inhabit. Their practices situate them outside the law, and both bakers frequently emphasized how contact with ministerial functionaries made their business hazardous and their livelihoods insecure. In addition, other bakers are not necessarily as successful in negotiating the fine lines between pragmatism, necessity, resourcefulness and bribes. Some are caught between government regulations and informal norms, unable, unwilling or unsuccessful at traversing this volatile terrain. Others face selective enforcement, repeated closures and the relentless pressure of ministerial prosecution.

Regardless of the vitality and pervasiveness of their efforts, both Samir and Hani went to great lengths to stress how the state apparatus maintains control over a basic subsistence good crucial to the citizenry, performing its power in the process.^75^ Of course, their efforts may ‘work back’ onto neoliberal and authoritarian logics, establishing limits on the penetration of the market and bureaucratic girds while demonstrating what alternative economic practices may look like.^76^ Yet at the same time, tacit toleration of such activities may help prevent broad-based protest and social unrest. This is why de Certeau emphasizes the flagrant ambivalence of tactics, they can be both “expansively inclusive and oppressively exclusive,” and need not come in emancipatory forms.^77^ What is clear, however, is that state interventions are always incomplete, unfinished projects, as the calculative practices of government allow for unforeseen reconfigurations. Dichotomies of domination/resistance, state/society and legality/illegality cannot fully capture the contingencies of everyday practice.

The payment of bribes to ensure subsidized flour, the stubborn use of crowd action to safeguard illegal sweets are not oriented against an abstract authoritarian regime or a unitary state, but are far more situated—enmeshed in “contingent constellations of practice, milieu and materiality.”^78^ Born of necessity and perpetuated by cooperation and compromise, these practices allow bakers to transform peripheral places within the urban landscape into arenas in which they can negotiate for services, ensure subsistence and maintain cohesive communities. Similar actions at any one of Amman’s 478 bakeries illustrate forms of agency that neither escape the state’s categories nor its constraints, but engage state power within the field in which it is exercised. De Certeau’s concept of “tactics” is useful in this respect, as it offers a less loaded view of people’s reluctance, refusal and negotiation in the face of power. It offers a more ambiguous, less decisive, less ideologically driven lens through which to analyse the many ways citizens inhabit their subordination. Samir and Hani’s actions are less about rebellion than calculated re-appropriation, the cultivation of modes of togetherness and alternative forms of exchange that make precarity and inter-dependence livable. As Mahmood argues, “While acts of resistance to relations of domination constitute one modality of action, they certainly do not exhaust the field of human action.”^79^ That is to say, agentic capacity is not only operative in those practices that contest domination but also in the various ways people inhabit power^80^ Yet far too often, we define political agency restrictively, as an active stance of defiance against domination that takes places within a demarcated public sphere or an intentionally concealed form of resistance to hegemonic norms. In doing so, we not only foreclose certain questions about the workings of power,^81^ but impose norms of hypervisibility and liberal politics on peoples who have different ways of making community and warding off control.^82^ To grasp this is to recognize the conundrums of those who live and labor in authoritarian settings and appreciate the vast political terrain that lies between quiescence and revolt. It is also to embed the politics of place in everyday life, not as simple sites of subaltern resistance nor as part of a governmental apparatus of acquiescence. In the Hashemite Kingdom, Amman’s bakeries are so much more.

## Footnotes

1 In order to preserve the anonymity of my informants and interviewees, whose ostensibly illegal activities I will describe herein, I have used pseudonyms for people and unspecified designations for the districts in which I conducted fieldwork: Jabal B and Jabal C.

2 As of February 2018, this price has been increased.

3 For more on how welfare provision in Jordan intersects with emergency aid for Syrian refugees, see José Ciro Martinez, “Bread is Life: The Intersection of Welfare Politics and Emergency Aid in Jordan,” *Middle East Report,* 44:272 (2014): 30-35.

4 During this time, I regularly participated in daily ‘office’ routines, usually during the early morning or late afternoon hours and always with the permission of bakery owners.

5 What, in Lefebvrian terms, would amount to l’espace *conçu, véçu* and *perçu.* For more on Lefebvre’s “tantalizingly vague” triad, see Andrew Merrifield, “Place and Space: A Lefebvrian Reconciliation,” *Transactions of the Institute of British Geographers,* 18:4 (1993): 516-531.

6 Tania Murray Li, *The Will to Improve: Governmentality, Development, and the Practice of Politics* (Durham, nc: Duke University Press, 2007), 126.

7 Michel Foucault, “Governmentality,” in *The Foucault Effect,* ed. Graham Burchell, Colin Gordon and Peter Miller (Chicago: University of Chicago Press, 1991), 103.

8 Mitchell Dean, *Governmentality: Power and Rule in Modern Society* (Sage: London, 1999), 2.

9 Matthew G. Hannah, *Governmentality and the Mastery of Territory in Nineteenth-Century America* (Cambridge: Cambridge University Press, 2000), 124; James C. Scott, *Seeing like a State: How Certain Schemes to Improve the Human Condition Have Failed* (New Haven: Yale University Press, 1998).

10 Bruno Latour, “The Powers of Association,” *The Sociological Review,* 32S1 (1984): 264-280.

11 Nikolas Rose, *Powers of Freedom: Reframing Political Thought* (Cambridge: Cambridge University Press, 1999), 36.

12 Peter Burke, *Social History of Knowledge: From Gutenberg to Diderot* (Cambridge: Polity Press, 2000); Thomas Lemke, *Foucault, Governmentality and Critique* (Boulder: Paradigm Publishers, 2011).

13 Thomas Biebricher, “Critical Theories of the State: Governmentality and the Strategic-Relational Approach,” *Constellations,* 20:3 (2013): 388-405.

14 Tanya Murray Li, “Governmentality,” *Anthropologica,* 49:2 (2007): 277. Foucault similarly emphasizes how life constantly escapes integration into the techniques of power that attempt to govern it. Michel Foucault, *The History of Sexuality. An Introduction. Volume 1* (New York: Vintage Books, 1990), 143.

15 Daniel Neep, “State-Space beyond Territory: Wormholes, Gravitational Fields, and Entanglement, Journal *of Historical Sociology* 30.3 (2017): 475.

16 Henri Lefebvre, *The Production of Space,* trans. Donald Nicholson-Smith (Oxford: Black-wel, 1991), 370. Neil Brenner and Stuart Elden, “Henri Lefebvre on State, Space, Territory,” *International Political Sociology,* 3:4 (2009): 358.

17 Empirical studies have shown the variety of administrative, legal and technical practices that permit the state apparatus to construct and disseminate abstract space. For an excellent summary, see Neep, “State Space Beyond Territory.”

18 Though cast through a familiar resistance/domination dichotomy, Lefebvre’ *Production of Space* (1991) makes clear how everyday life is imbued with power, never neatly responding to the ordered diagrams and static distributions of the state apparatus.

19 Elden and Brenner, “Henri Lefebvre,” 367.

20 Henri Lefebvre, as quoted in Andrew Merrifield, *Henri Lefebvre: A Critical Introduction* (New York: Routledge, 2006), 10.

21 Michel de Certeau, *The Practice of Everyday Life* (Berkeley: University of California Press, 1984), xiv. For useful accounts of the difference between strategy and tactics, see Ian Buchanan, *Michel de Certeau: Cultural Theorist* (London: Sage, 2000), 86-108; Ben Highmore, *Everyday Life and Cultural Theory: An Introduction* (New York: Routledge, 2002), 145-174.

22 de Certeau, *The Practice of Everyday Life,* xxiv; Donald S. Moore, “Subaltern Struggles and the Politics of Place: Remapping Resistance in Zimbabwe’s Eastern Highlands,” *Cultural Anthropology,* 13:3 (1998): 350.

23 de Certeau, *The Practice of Everyday Life,* 26 and 37.

24 James C. Scott, *Weapons of the Weak: Everyday Forms of Resistance* (New Haven: Yale University Press, 1985); James C. Scott, *Domination and the Arts of Resistance: Hidden Transcripts* (New Haven: Yale University Press, 1990).

25 Reinoud Leenders and Steven Heydemann, “Popular Mobilization in Syria: Opportunity and Threat, and the Social Networks of the Early Risers,” *Mediterranean Politics* 17:2 (2012): 139-159; Charles Tripp, *The Power and the People: Paths of Resistance in the Middle East* (Cambridge: Cambridge University Press, 2013); Sophie Richter-Devroe (2011) “Palestinian Women’s Everyday Resistance: Between Normality and Normalisation,” *Journal of International Women’s Studies,* 12:2 (2011): 32-46; Salwa Ismail, “Urban Subalterns in the Arab Revolutions: Cairo and Damascus in Comparative Perspective,” *Comparative Studies in Society and History* 55:4 (2013): 865-894.

26 Salwa Ismail, *Political Life in Cairo’s New Quarters: Encountering the Everyday State* (Minneapolis: University of Minnesota Press, 2006), xxiii.

27 Asef Bayat, *Life as Politics: How Ordinary People Change the Middle East* (Palo Alto: Stanford University Press, 2010).

28 Jonathan Shapiro Anjaria, *The Slow Boil: Street Food, Rights and Public Space in Mumbai* (Palo Alto: Stanford University Press, 2016), 105.

29 Lila Abu-Lughod, “The Romance of Resistance: Tracing Transformations of Power through Bedouin Women,” *American Ethnologist,* 17:1 (1990): 41-55.

30 Sherry B. Ortner, “Resistance and the Problem of Ethnographic Refusal,” *Comparative Studies in Society and History,* 37:1 (1995): 173-193.

31 Judith Butler, *Notes Toward a Performative Theory of Assembly* (Cambridge: Harvard University Press, 2015), 218; Amira Mittermaier, “Bread, Freedom, Social Justice: The Egyptian Uprising and a Sufi Khidma,” *Cultural Anthropology,* 29:1 (2014): 54-79; Laleh Khalili, “The Politics of Pleasure: Promenading on the Corniche and Beachgoing,” *Environment and Planning D: Society and Space* 34.4 (2016): 583-600.

32 For more on protective welfare states, see Nita Rudra, “Welfare States in Developing Countries: Unique or Universal?” Journal *of Politics,* 69:2 (2007): 378-396.

33 Anne Marie Baylouny, *Privatizing Welfare in the Middle East: Kin Mutual Aid Associations in Jordan and Lebanon* (Bloomington: Indiana University Press, 2010), 168.

34 “Jordan: New Government,” *Oxford Analytica Daily Brief,* 2 May 1989.

35 Baylouny, *Privatizing Welfare,* 55.

36 Curtis Ryan, “Peace, Bread and Riots: Jordan and the International Monetary Fund,” *Middle East Policy,* 6:2 (1998): 54-66.

37 John Chalcraft, *Popular Politics in the Making of the Modern Middle East* (Cambridge: Cambridge University Press, 2016), 532; Larbi Sadiki, “Popular Uprisings and Arab Democratization,” *International Journal Middle East Studies,* 32:1 (2000): 71-95.

38 José Ciro Martinez, “Leavened Apprehensions: Bread Subsidies and Moral Economies in Hashemite Jordan,” *International Journal of Middle East Studies* 50:2 (2018): 173-193; José Ciro Martinez, “Jordan’s Self-fulfilling Prophecy: The Production of Feeble Political Parties and the Perceived Perils of Democracy,” *British Journal of Middle Eastern Studies* 44.3 (2017): 356-372.

39 Kevin J. O’Brien and Lianjiang Li, *Rightful Resistance in Rural China* (Cambridge: Cambridge University Press, 2006).

40 Najib B. Hourani (2014) “Neoliberal Urbanism and the Arab Uprisings: A View from Amman,” *Journal of Urban Affairs,* 36:s2 (2014): 650-662; Eliana Abu-Hani, “Neoliberalism as a Site-Specific Process: The Aesthetics and Politics of Architecture in Amman, Jordan,” *Cities,* 60:A (2017): 102-112.

41 Anne Mariel Peters and Pete W. Moore, “Beyond Boom and Bust: External Rents, Durable Authoritarianism, and Institutional Adaptation in the Hashemite Kingdom of Jordan,” *Studies in Comparative International Development* 44:3 (2009): 256-285; Abu-Hani, “Neoliberalism as a Site-Specific Process,” 102-112.

42 Baylouny, *Privatizing Welfare,* 75-78.

43 Baylouny, *Privatizing Welfare,* 78.

44 Asef Bayat, “Activism and Social Development in the Middle East,” *International Journal of Middle East Studies* 34:1 (2002): 1-28.

45 Wendy Wolford, “Families, Fields, and Fighting for Land: The Spatial Dynamics of Contention in Rural Brazil,” *Mobilization,* 8:2 (2003): 157-172; Erica Simmons, *Meaningful Resistance: Market Reforms and the Roots of Social Protest in Latin America* (Cambridge: Cambridge University Press, 2016).

46 Simmons, *Meaningful Resistance,* 9.

47 Steven L. Kaplan, *The Bakers of Paris and the Bread Question, 1700-1755* (Durham, NC: Duke University Press, 1996).

48 For one recurring example, see Dana Al Emam (2015) “Bakery Owners Expect Production Slowdown in Eid over ‘Flour Shortage’,” *Jordan Times* 29 June, http://wwwjordan times.com/news/local/bakery-owners-expect-production-slowdown-eid-over-flour-shortage%E2%8o%99.

49 Finn Stepputat, (2013) “Contemporary Governscapes: Sovereign Practice and Hybrid Orders Beyond the Center,” in *Local Politics and Contemporary Transformations in the Arab World,* ed., Malika Bouziane, Cilja Harders and Anja Hoffman (London: Palgrave Macmillan, 2013), 31.

50 Literally, this phrase means to pay before you raise. What exactly is being raised is purposely left ambiguous. This phrase is used in various idiomatic sayings. One is common to public protests, in which demonstrators request payment (symbolic, or in the way of a material transfer) before price increases. In the context in which it is uttered here, it is analogous to a common expression I heard used by single young men, in which you must pay (al-daf’a) before (qabal) raising (al-raf’a), a women’s skirt (often in reference to a prostitute). An alternative translation would be to pay in advance. I have chosen “pay to play,” as I feel it is the most accurate rendition in this context.

51 Most purchases of subsidized flour on the black market are destined to the production of sweets and biscuits with higher profit margins than standard *khubz arabl.*

52 While exploring the use of Islamic vocabularies is beyond the scope of this paper, various scholars have analyzed how religious frames are mobilized to critique prevailing political and economic arrangements. Cihan Tugal, “Transforming Everyday Life: Islamism and Social Movement Theory,” *Theory and Society,* 38:5 (2009): 423-458; Charles Tripp, *Islam and the Moral Economy: The Challenge of Capitalism* (Cambridge: Cambridge University Press, 2006); Yael Navaro-Yashin (1998) “Entrapped Between Categories: ‘East’, ‘West’ and the Practices of Consumption of Turkish Islamists,” *Sociologus* 48:1 (1998): 1-16.

53 David Harvey, *The Enigma of Capital, and the Crises of Capitalism* (London: Profile Books, 2010), 26.

54 Tacit knowledge of Hani’s weekly bribe resembles what Taussig describes as a “public secret,” widely known but not spoken about publicly. Michael T. Taussig, *Defacement: Public Secrecy and the Labor of the Negative* (Palo Alto: Stanford University Press, 1999).

55 Jonathan Shapiro Anjaria, “Ordinary States: Everyday Corruption and the Politics of Space in Mumbai,” *American Ethnologist* 38:1 (2011): 67.

56 Moya Kneafsey et al., *Reconnecting Consumers, Producers and Food: Exploring Alternatives* (Oxford: Berg, 2008).

57 A large sheet of unleavened bread, it is far thinner and more difficult to prepare than khubz ’arabl.

58 For more on mansaf, see Sally Howell, “Modernizing Mansaf: The Consuming Contexts of Jordan’s National Dish,” *Food & Fooctways,n:4* (2003): 215-243.

59 Varieties of flour are distinguished by their extraction rate, the amount of bran and germ left in the flour after milling. *Al-Muwahhad* has a 78% extraction rate, while other varieties typically lie somewhere between 55-68%.

60 During most of 2013 subsidized flour cost Jordan’s bakers 35 *dinars* per ton ($49 USD), while the international market price hovered around 301.5 *dinars* ($426 USD).

61 The boa is the professional association charged with representing the interests of bakery owners in Jordan.

62 Samir’s account is confirmed by various reports published at the time. For details on the 2007 reform, see Ala al-Furwati (2007) “Darabka fi al-makāhbiz wa ghadab fi al-shāri’a b’ad raf’a al-d’am ’an al-tahin,” *As-Sijil* 2 (15 November), http://www.al-sijill.com/sijill_items/sitem86.htm.

63 This information is based on thirty-four interviews with various employees of the Ministry of Industry, Trade and Supply, conducted between October 2013 and December 2015.

64 Anjaria, *The Slow Boil,* 126.

65 Lisa Mitchell, “’To stop train pull chain:’ Writing Histories of Contemporary Political Practice,” *Indian Economic & Social History Review* 48:4 (2011): 469-495.

66 Anjaria “Ordinary States,” 85; AbdouMaliq Simone. *City life from Jakarta to Dakar: Movements at the Crossroads.* London: Routledge, 2010.

67 Lisa Bjorkman, “The Ostentatious Crowd: Public Protest as Mass-Political Street Theatre in Mumbai,” *Critique of Anthropology,* 35:2 (2015): 142-165.

68 Giorgio Blundo and Jean-Pierre Olivier de Sardan, *Everyday Corruption and the State: Citizens and Public Officials in Africa* (London: Zed Books, 2006), 5.

69 Diane Singerman, *Avenues of Participation: Family, Politics, and Networks in Urban Quarters of Cairo* (Princeton: Princeton University Press, 1995), 14; Partha Chatterjee, *The Politics of the Governed: Reflections on Popular Politics in Most of the World* (Columbia University Press, 2015); José Ciro Martinez, “Leavening Neoliberalization’s Uneven Pathways: Bread, Governance and Political Rationalities in the Hashemite Kingdom of Jordan,” *Mediterranean Politics,* 22:4 (2017): 464-483.

70 Anjaria, *The Slaw Boil,* p. 8.

71 Ortner, “Resistance and the Problem of Ethnographic Refusal,” 173-193.

72 Nicos Poulantzas, *State, Power, Socialism* (London: Verso, 1973); Akhil Gupta, *Red Tape: Bureaucracy, Structural Violence and Poverty in India* (Durham, NC: Duke University Press, 2012), 108-109.

73 Anjaria, *The Slaw Boil,* 18-19.

74 For more on the pragmatism-radicalism dialectic in the study of local politics which informs these two interpretations, see Paul Amar, “Beyond the ‘Pragmatism-Radicalism Dialectic’ in the Study of Local Politics,” in *Local Politics and Contemporary Transformations in the Arab World,* ed. Malika Bouziane, Cilja Harders and Anja Hoffman (London: Palgrave Macmillan, 2013), 65-90.

75 For more on how the provision of bread can function as a crucial performance of state authority, see José Ciro Martinez and Brent Eng, “Struggling to Perform the State: The Politics of Bread in the Syrian Civil War,” *International Political Sociology* 11.2 (2017): 130147; José Ciro Martinez and Brent Eng, “Stifling Stateness: The Assad Regime’s Campaign against Rebel Governance,” *Security Dialogue* (2018), http://journals.sagepub.com/doi/abs/10.1177/0967010618768622.

76 Adrian Smith, “Articulating Neo-liberalism: Diverse Economies and Everyday Life in ‘Post-Socialist’ Cities,” in *Contesting Neoliberalism: Urban Frontiers,* ed. Helga Leitner, Jamie Peck, and Eric S. Sheppard (New York: Guilford Press, 2007), 204-222; *Bayat, Life as Politics,* 249.

77 Ben Highmore, *Michel de Certeau: Analysing Culture* (London: Continuum, 2006), 114.

78 Donald S. Moore, *Suffering/or Territory:Race, Place, and Power in Zimbabwe* (Durham, NC: Duke University Press, 2005), 44.

79 Saba Mahmood, *Politics of Piety: The Islamic Revival and the Feminist Subject.* Princeton: Princeton University Press, 2012), x.

80 Ibid., 15.

81 Abu-Lughod, “The Romance of Resistance,” 41-55.

82 Butler, *Notes Towards a Performative Theory,* 56; Mahmood, *Politics of Piety,* 9-14.

